# INTEGRATING ANIMAL MODEL, HUMAN SUBJECTS, AND SOCIAL SYSTEMS IN NUTRITION AND AGING RESEARCH

**DOI:** 10.1093/geroni/igad104.0288

**Published:** 2023-12-21

**Authors:** Sarah Booth, Haowei Wang, Roger Fielding

## Abstract

This symposium will bring together speakers who will present cutting-edge research on nutrition and aging from interdisciplinary perspectives using complementary methods. Nutrition plays an important role in understanding the biology of aging, promoting healthy aging, preventing age-related diseases, and supporting aging populations at risks. This symposium will present innovative studies using animal models, population surveys, and nutrition programs to inform future research and promote nutrition security and diet quality. Lee et al. examine sex-specific differences in gut homeostasis and host metabolism in female and male mice fed different rodent diets. Shea et al. and Ardisson Korat et al. focus on dietary quality and patterns in older populations. Using a novel network analysis, Shea et al. examine similarities and differences in the manner in which food groups are consumed in a nationally representative sample from the National Health and Nutrition Examination Survey. Ardisson Korat et al. utilize data from the Nurse’s Health Study and evaluate the long-term role of dietary carbohydrate quality in promoting healthy aging. Borth & Whitmire and El-Abbadi et al. investigate food insecurity and nutrition programs among older adults during and after the COVID-19 pandemic. Using data from US Census Bureau’s Household Pulse Survey (April 2020 to December 2022), El-Abbadi et al. explore the differences in food insecurity by age-groups, gender, and race and ethnicity. Borth & Whitmire conduct research on congregate nutrition programs to identify successful practices of nutrition programs during the pandemic and discuss challenges and directions in future congregate nutrition programs. This is a Nutrition Interest Group Sponsored Symposium.

